# Report on the Progress of Forensic Medicine

**Published:** 1846-05

**Authors:** Wm. Augustus Guy

**Affiliations:** M.B., Cantab.; Fellow of the Royal College of Physicians, Professor of Forensic Medicine, King’s College, Physician to the King’s College Hospital, &c.


### SELECTIONS

FROM

### AMERICAN AND FOREIGN JOURNALS.

---

*Report on the Progress of Forensic Medicine.*—By Wm. Augustus Guy, M.B., Cantab. Fellow of the Royal College of Physicians, Professor of Forensic Medicine, King's College, Physician to the King's College Hospital, &c.

### TOXICOLOGY.

*Poisoning by Hydrocyanic Acid.*—The subject of poisoning by hydrocyanic acid holds the same place, in point of importance, as it did in the former report. No less than five cases have been recorded in the English journals during the last half of the year. Of four of these it is proposed to give a full account, adding such additional facts and observations as can be gleaned from the cases themselves, or from the commentaries which accompany them.

*Two Cases of Poisoning by Prussic Acid, by Mr. Nunneley, of Leeds.*—Two interesting, instructive, and minutely detailed cases of poisoning by this acid, were communicated to the Provincial Medical and Surgical Journal, by Thomas Nunneley, of Leeds. The particulars of the first case are condensed from the evidence given before the inquest held upon the body; those of the second case, which terminated favorably, chiefly the man's own account.

The leading particulars of the first case, as gleaned from Mr. Nunneley's communication, are as follows:

The deceased purchased of a druggist an ounce of prussic acid, labelled 'Scheele's strength,' but which was subsequently proved by careful analysis to contain only 1·5 per cent. Of this it would seem *probable*, that five drachms and a half were swallowed by the deceased, as the bottle when found on his person contained two and a half drachms. The circumstances under which the poison was swallowed are involved in some obscurity; but from a comparison of all the facts detailed in evidence, or observed by Mr. Nunneley himself, it is most probable that the deceased poured the poison into a tumbler which he found on the table of a room on the ground floor, swallowed it, rinsed the glass out, threw the water used for this purpose into and about a spittoon, replaced the stopper in the bottle, and put the bottle into his pocket, ran up stairs with the tumbler to a room on the floor above, placed the tumbler on a table, and then threw himself on the sofa at the end most remote from the part of the table on which he had set the glass. About three minutes from the time the deceased was heard to enter the lower room, he was found by one of the witnesses (Mr. Tennant) lying on his back on the sofa, but as soon as he heard him enter the room, 'he sprang up, and sat upon-an-end.' He did not speak, but appeared like a man who was suffering from an excess of drink. Mr. Tennant then left the room, and returned almost immediately, when he found the deceased sitting in the same position, with the exception that he was reclining on the right side, with his head on the sofa cushion. Mr. Tennant then said, 'You seem ill, sir,' when he made an attempt to speak, but was not intelligible. The witness then went close up to him, and said, 'You must excuse my interfering with you, but you seem very ill,' and then asked if he could be of any assistance to him, if he might fetch a doctor. The deceased in reply laid his hand on his breast, and said, 'Oh no, it is too late, it is too late.' These were the only words he uttered. Mr. Tennant then rang the bell for the landlady who attended the deceased, while Mr. Tennant ran for a medical man. In her evidence she stated, that she thought the deceased was in a fit, and accordingly loosed his handkerchief and collar, and bathed his head with brandy. In about five minutes from Mr. Tennant's leaving the room, and about a quarter of an hour from the time that the deceased was heard to enter the house, Mr. Nunneley arrived. He found the deceased still lying on the sofa, resting on his right side, unable to speak, and apparently unconscious, but the pulse was still beating slowly, but distinctly. On reaching the top of the stairs, near the door of the room, Mr. Nunneley thought he detected the smell of hydrocyanic acid, but on going to the end of the room where the deceased was, he could not detect it in consequence of the strong odor of the brandy, which had been used to bathe the head of the deceased. Suspecting that the deceased had taken hydrocyanic acid, no time was lost in dashing cold water upon him, which 'decidedly roused him.' This remedy was repeated with effect several successive times; 'it roused him, and he took a deep inspiration.' Ammonia was also administered, and freely applied to his chest. A small quantity of a solution of chlorine was also given; and the feet and legs placed in a hot mustard bath. The deceased was conscious of the administration of the ammonia. In spite of these remedies, however, 'he gradually became stiller, and sank; his legs, arms, and chest were slightly convulsed, the upper extremities more than the lower, and the jaw was fixed; the eyes were prominent and staring, and the pupils widely dilated; but before death they became much less so; they were glassy. The countenance was puffed up and dark, rather purple. There was some foam about the mouth; he breathed slowly and convulsively, something like violent sobbing. The blood-vessels were injected, and the whole of the countenance bloated. The hands were partially contracted, the fingers rigid, and about the nails of a dark lead color.' The time which elapsed between the arrival of Mr. Nunneley and the death of the patient amounted to about half an hour; it therefore follows, that he survived the taking of the poison three-quarters of an hour.

The following is a short abstract of the post-mortem appearances presented by the body five hours and a half after death:

*External appearances.*—Body still warm; upper parts pallid, but all the depending portions livid. the cutaneous veins, especially those of the legs, filled with fluid blood; the limbs about as rigid as is usual at the same period after death; the fingers, at the root of the nails discolored; the countenance placid, but somewhat turgid; the eyelids closed; the eyes prominent and glassy; the pupils moderately dilated; the jaws contracted, and the lips closed. 'There was the odor of hydrocyanic acid in the room near the body. The degree, however, in which this could be perceived by those present, on applying in succession the nose to the nostrils of the deceased, differed materially; by Dr. P. Smith very strongly; by myself decidedly, but not what I should call strongly; and by the other two gentlemen, scarcely, if at all; indeed, they could not say they did perceive it.' Elsewhere, Mr.

Nunneley says, 'It (the odor) was perceptible to my nostrils even before the body was opened. It was so strong that I had an effect from it for some hours afterwards; that is, constriction about the pharynx.'

*Abdomen.*—On opening the peritoneum, the odor of hydrocyanic acid was instantly perceptible in any part of the room; but most distinctly close to the stomach. All the viscera healthy. No odor of the acid in the intestines; but a sour smell, with an exceedingly strong odor of the acid in the contents of the stomach. The stomach contained rather more than a pint of half digested matter (he had eaten a hearty breakfast about two hours and a half before taking the acid), and its mucous membrane exhibited the turgescence of the vessels usual during the process of digestion.

*Chest.*—Lungs healthy; large bronchial tubes filled with reddish frothy serum. Heart healthy, but completely distended on both sides with dark fluid blood.

*Head.*—Chronic inflammation of the arachnoid; serous effusion on the surface and into the ventricles; substance of the brain firm, perhaps rather more so than usual.

The *chemical analysis* was performed by Mr. West. The substances submitted to analysis were the contents of the stomach, the matters contained in about fifteen inches of the ileum, two ounces of blood from each side of the heart, and three drachms of serum from the brain.

*Contents of the Stomach.*—Strong acid reaction; marked odor of prussic acid; decided reaction with the silver and iron tests; four-tenths of a grain of pure hydrocyanic acid; equal to twenty-seven grains of medicinal acid of 1·5 per cent. (the strength of the acid in the bottle), were obtained from the contents. From experiments on the contents of the ileum, the blood and the serum of the brain, Mr. West states that he thought himself 'warranted in stating, the arterial blood, and the fluid from the brain, as well as the contents of the ileum, to contain a distinct, though very slight trace of hydrocyanic acid; the venous blood none.' This fact is inferred from the production of a film upon a drop of nitrate of silver inverted over the substances in question. The products of the distillation of the contents of the ileum precipitated nitrate of silver, but did not yield Prussian blue: the liquid distilled from the arterial blood and serum, neither affected the silver test, nor yielded Prussian blue. It is obvious therefore, that the presence of hydrocyanic acid in these fluids must be considered to be full as doubtful as the passage just quoted would make it. The parties present thought that they perceived the odor of prussic acid in the serum from the ventricles of the brain; 'Mr. Nunneley with some hesitation, but still he thought he could perceive it; Dr. P. Smith perceived it so decidedly as to have no hesitation about it.'

Such is a careful abstract of this extremely interesting case. Some observations communicated by Mr. Nunneley to the succeeding number of the same journal, will be noticed in their proper place.

The second case of poisoning by this acid reported by Mr. Thomas Nunneley will be found in the Provincial Medical and Surgical Journal for August 13th, 1845. When Mr. Nunneley saw the patient the symptoms had passed away, but 'he was able to give a detailed and perfectly natural account of the whole transaction, and of his feelings at the time of becoming insensible. This he repeated the next day, and the different parts of it were so confirmed by different persons, that there cannot be any doubt of its accuracy.' The patient, a book-keeper in a large manufacturing establishment, 40 years of age, bought three-penny worth of prussic acid under the pretence of poisoning animals, and on going to bed at night undressed himself by the bedside, nearly filled a wine-glass with gin, emptied the bottle containing the hydrocyanic acid into the glass, corked the bottle, and placed it on the chimney-piece, drank off the contents of the glass, which he set down, then threw down the bed-clothes, got into bed, and covered himself up. He now said to his wife, 'Well, Bessy, I have taken something. and it will be all over with me in a few minutes.' She immediately jumped out of bed, and ran screaming to the door. When she left the bed, the child, who was frightened, crept to its father, and said to him, as he informed Mr. Nunneley, 'Father, don't leave me;' to which he replied, putting his arms around it, 'No, my dear, I won't leave you.' He now perceived his jaws becoming stiff, and crammed the sheet between his teeth, thinking, as he said, that he would like something to hold by: he felt his jaws becoming stiffer and tighter, which was all he was conscious of until the water was dashed in his face, when he raised himself, and to his surprise, found several persons round the bed. He had no idea how long he had been unconscious, and said that he felt no pain till the water was dashed upon his face; the sensation he then felt, he said, was most awful, he could not describe it. About two minutes from the time the poison was taken the wife returned, and found her husband lying upon his back, insensible, breathing hard, the eyes wide open and staring, the face 'black red.' In about ten minutes from the swallowing of the poison Mr. Bishop reached the house, and found the man foaming at the mouth, the face rather pale, and covered with large drops of sweat, the arms rigid, the hands half extended, the jaws so firmly closed as to prevent him from giving an emetic of sulphate of zinc. Mr. Bishop immediately dashed cold water over the head and neck, when the man raised himself up and became sensible; ammonia was then rubbed over the hands, the smell of which he quickly perceived, and said he felt better for it, and the sulphate of zinc was administered; vomiting soon followed, the matter ejected smelling strongly of hydrocyanic acid. About a pint of blood was next taken from the arm, which had a muddy-red brick tint, and coagulated as usual. There was a difference of opinion as to the blood having an odor of prussic acid, but the poison could not be detected in it by chemical tests. The strength of the acid was found to be three and a quarter per cent. of real acid. The quantity of acid taken was estimated at 40 drops, so that the man must have taken nearly one grain and a half of anhydrous prussic acid. Twenty minims of the same pre-preparation destroyed a small half-grown mongrel dog in two minutes and a half, and a full-sized terrier bitch in one minute.

A report of the same case by Mr. Edward Bishop, surgeon of Kirkstall, agreeing in all essential particulars with the foregoing, will be found in the Lancet for 20th of September, 1845.

*Two Cases of Poisoning by Hydrocyanic Acid, by Mr. Hicks, of Newington.*—Mr. James Hicks of Newington, has made an important addition to our knowledge of the effects of prussic acid, by the publication of two cases, accompanied by some judicious comments, in the pages of the Medical Gazette. The first case is an abridgement of one published in a former number of the Gazette; the second is related for the first time, from memory. The following are the leading particulars of the first case. A female, about 22 years of age, swallowed a draught containing, as ascertained by analysis, *nine-tenths* of a grain of pure anhydrous acid. It appears from the statement of the father, who was present when the medicine was taken, that, on swallowing it, the girl started up, threw her hands over her head, drew a deep gasping breath, stood for a second, and, then, running forward for a short distance with great violence, fell with her head first to the ground, after which she never moved, but continued to make a moaning noise for five minutes afterwards. On Mr. Hicks's arrival he found the girl lying on her back, perfectly insensible, with her teeth clenched, foam issuing from her mouth, and the face so greatly congested as to be almost purple; the breathing was slow, laborious, and at long intervals; the pulse imperceptible, and the action of the heart indistinct; the eyelids were partly closed, the eyes appearing as if pushed forward between them, the pupils fully dilated and insensible to light; the body was so strongly convulsed that the head seemed buried between the shoulders, and the arms were nearly turned round by the action of the pronator muscles. Mr. Hicks's first impression was, that the patient was laboring under some form of epilepsy; but having ascertained that the symptoms came on immediately after taking a dose of medicine, he was soon convinced, by the taste and smell of the liquid, that she had taken prussic acid. Ammonia was accordingly applied to the nostrils, the cold affusion to the head, and a fruitless attempt was made to administer some brandy and ammonia. In spite of this treatment 'the breathing became gradually slower, and at longer intervals, until it entirely ceased, death appearing to have been caused, in this instance, by the perfect inability of the patient to get air into the lungs by inspiration; the time that had elapsed between taking the poison and death being twenty minutes.

The body was examined ninety hours after death, and presented the following appearances. Externally it had much the appearance of that of a person who had died of asphyxia; the teeth were clenched, the mouth covered with foam, the face of a dusky red hue, and the whole of the depending parts of the body of a dark purple or violet color. The internal appearances were as follows:

*Head.*—The dura mater and sinuses much congested, the whole substance of the brain dotted with fluid black blood, the ventricles empty, and the plexus choroides pale and bloodless. No odor of prussic acid perceptible.

*Chest.*—Odor of prussic acid distinctly perceptible, far more so than in any other part of the body; lungs healthy, but much congested; the right ventricle of the heart loaded with blood; the aorta empty; the blood fluid and very black.

*Abdomen.*—Stomach containing about four ounces of fluid, smelling strongly of prussic acid; its lining membrane, with the exception of a small red patch on the posterior part, near the cardiac orifice, healthy; the kidneys much congested, and of a dark pinkish hue; the rest of the viscera healthy.

The contents of the stomach were examined five days after death. The odor of prussic acid was still perceptible, and the poison was detected by the iron and silver test. It was ascertained that the quantity of the poison taken did not exceed *nine-tenths* of a grain of pure anhydrous acid, a quantity equivalent to 18 grains of Scheele's acid, or 45 of the pharmacopœial acid, the former of which contains five per cent., and the latter two per cent.

A report of this case, differing but little from that published in the Medical Gazette, was communicated by Mr. Hicks to the Lancet, and is followed by a short account of the same case drawn up by Mr. Watson and communicated by Dr. Letheby. The only discrepancies which it is necessary to point out are the following:

1. The prussic acid lotion, which was taken by mistake, contained, according to Mr. Hicks, three grains and a half of anhydrous acid; but according to Dr. Letheby's analysis, four grains. The fourth part, therefore, would contain, according to the first estimate, rather less than nine-tenths of a grain; and, according to the second, one grain.

2. Mr. Hicks states that death took place in twenty minutes; Dr. Letheby, on the authority of Mr. Watson, 'just fifteen minutes from the time of her taking the medicine.'

3. In addition to the statement that the odor of the prussic acid was distinctly perceptible, in the chest, we are told that 'this was still more marked in the fluid contained in the pericardium.'

4. The chief discrepancy, however, is to be found in the description of the stomach and its contents. Dr. Letheby says, 'the stomach contained two ounces of a thick whitish fluid, apparently composed of biscuit and water. Neither of the three gentlemen whom I have named (Messrs. Watson, Waterworth, and Hicks), could detect any odor of prussic acid; but it was evident to myself, even on the following day. On subjecting it, however, to a chemical analysis, I could but barely recognise the presence of this poison. The interior of the stomach presented those marks of ecchymosis which I have noticed and described in my three former cases.' The following facts are necessary to complete the case. The deceased did not speak after taking the poison. She was not sick; nor were the fæces or urine expelled.

The absence of the shriek or scream has been already noticed.

Mr. Hicks's second case is as follows: 'A girl having quarrelled with her lover, expressed a wish to leave the room, on account, as she stated, of feeling faint; but she had not done so more than a minute when she was observed to throw her hands over her head, and then fall to the ground; the breathing for a time was quite imperceptible, when, after making a few gasps for breath, she died five minutes after taking the poison, without having been in the least convulsed from the first. Within a short time after she died I arrived at the house; at this time the body was still warm, but, with the exception of a pallid state of the countenance, there was not an unnatural appearance to be seen about the corpse. It appears in this case that the girl took the poison as soon as she had left the room, and that she merely complained of feeling faint as an excuse for doing so. But the most interesting facts were, that, notwithstanding the dose was large, being an ounce of Scheele's, she had sufficient time to put the bottle into the front of her dress prior to volition being destroyed, while, at the same time, there were no convulsions, neither was there any scream, the girl falling down without making any noise whatever.'

*Case of Poisoning by Prussic Acid, by Dr. Guy.*—This case, of which the leading particulars are stated below, is interesting, first, by showing that a considerable interval of perfect consciousness, and complete command over the voluntary muscles, may intervene between the swallowing of a large dose of the acid and the development of its characteristic effects; and, secondly, as an instance of attempted suicide, in which the impulse to the commission of the act preceded the act itself by a very short interval of time.

There are some few facts of importance established by the foregoing cases which deserve to be briefly considered. This will be best done under distinct heads.

Symptoms.—*The Shriek.*—The preceding cases give no confirmation to the importance attached to the shriek as a symptom of poisoning by prussic acid. In Mr. Nunneley's fatal case no shriek was heard, and it is highly improbable from the facts detailed in evidence that any could have been uttered. The shriek was also absent in Mr. Nunneley's second case, in both the cases reported by Mr. Hicks, and in

Dr. Guy's case. It is very difficult, therefore, to understand whence the strong impression which has existed as to the importance of this symptom could have arisen. It is certainly not from observation in the human subject, unless indeed the exclamations uttered in one or two reported cases, or the loud gasping inspirations observed in other instances, have been confounded with a scream or shriek; so far from the shriek being a common symptom, Mr. Hicks says, 'Although I have read over every case published in the different works on poison and medical jurisprudence, I have not found a single instance where a shriek has been mentioned as occurring prior to death.' In animals, moreover, the shriek, though of more frequent occurrence than in the human subject, is by no means a constant symptom. Even when, as in the case of Mrs. Belaney, a shriek is reported to have been uttered, it is by no means satisfactorily made out that the scream or shriek was a symptom of poisoning by prussic acid. It is fully as reasonable to suppose that in her case it was a simple expression of alarm on finding that she had swallowed some of 'that hot drink,' of the fatal properties of which she was probably not ignorant. In the case of Sarah Hart, the noise which alarmed her neighbor was not a shriek, but 'a moan or stifled groan,'—probably the loud gasping inspiration which was so marked a symptom in Dr. Guy's case.

*Acts of Volition.*—The foregoing cases form a very useful addition to our information on this subject. In Mr. Hicks's second case the girl threw her hands over her head, fell to the ground, and after a few gasping respirations died in five minutes. The only act of volition consisted in placing the bottle in the front part of her dress. His first case was marked by strong muscular contractions but by no act of volition. The girl started up, threw her hands over her head, drew a deep gasping breath, stood for a second, and then, running forward for a short distance with great violence, fell with her head first to the ground, after which she never moved, but continued to make a murmuring noise for five minutes afterwards. She died at the end of twenty minutes. The other three cases were characterized by the performance of several acts of volition. After a dose of prussic acid which under less favorable circumstances would probably have proved rapidly fatal, the young lad, whose case is recorded by Dr. Guy, after swallowing the poison got out of bed, walked round the foot of the bed to a chest of drawers at some little distance from it, placed the stopper very firmly in the bottle, laid it on the drawers, walked back to bed, sat on the side of it, and then for the first time lost all consciousness. Mr. Nunneley's cases are still more remarkable, as the patients not only moved about, but spoke and answered questions after taking the one a fatal, the other a large, dose of prussic acid. The two cases were among the most remarkable and instructive yet recorded. For the several acts of volition performed, the reader is referred to the cases themselves. The fact, that both patients enjoyed the use of speech for some little time after taking the poison, is well worthy of remark. The subject of the first case answered a question addressed to him some minutes after he had swallowed the poison, and the man who recovered retained the use of his speech till the jaws gradually closed. If these facts had been known to the profession before the trial of Belaney, the medical witnesses would not have expressed themselves in such decided terms as to the improbability of voluntary motion or speech after the utterance of the shriek, especially if they had formed the very feasible opinion just expressed, that the shriek might not, after all, have been a symptom of the poisoning, but merely an expression of terror uttered during the interval of perfect consciousness and self-command which so often follows immediately on the swallowing of the poison.

*Duration.*—In Mr. Nunneley's first case, the duration, as far as it could be ascertained, was three-quarters of an hour; in Mr. Hicks's first case fifteen or twenty minutes, in his second case five minutes.

*Dose in Fatal Cases. Smallest Fatal Dose.*—In Mr. Nunneley's fatal case, the quantity could not be ascertained. It is probable, however, that it amounted to four drachms and a half of acid containing one and a half per cent. of pure anhydrous acid. One ounce had been purchased, and the bottle when found contained two drachms and a half. At any rate, this case throws no light on the question of the smallest dose which may prove fatal. In Mr. Hicks's first case the fatal dose did not exceed *nine-tenths* of a grain of anhydrous acid, or, according to Doctor Letheby's analysis, one grain.

*Quantity taken in Cases of Recovery.*—In Mr. Nunneley's second case, the quantity of anhydrous acid taken was estimated by Mr. West at one grain and a half. In Dr. Guy's case, neither the quantity nor the strength of the preparation could be ascertained.

Treatment. *Cold Affusion.*—'Of all the remedies I am acquainted with,' says Mr. Nunneley, 'I should be disposed to place most reliance upon cold affusion. In this instance (the fatal case) more effect was produced by the dashing of cold water than by any other means.' By way of illustration of the value of this remedy, Mr. Nunneley gives the following: 'Some years ago I well recollect taking a large Newfoundland dog to the side of a pond, and giving him upwards of a drachm of hydrocyanic acid, originally of Scheele's strength, but having been kept some time, it had probably lost something of its strength, yet, I am convinced, was sufficiently potent to destroy life. He almost immediately began to stagger, when two of us seized him by the legs, and threw him into the water, in which he swam about for a time, and presently came out, not much the worse for the dose.

*Tests.*—Mr. Nunneley, in citing Mr. West's analysis of the contents of the stomach in the fatal case reported by him, impugns the accuracy of the statement made in toxicological works, that the cyanide of silver is insoluble in cold, though soluble in boiling nitric acid. Dr. West says, 'I applied the acid, cold, to the washed precipitate, and found it to dissolve without heat. I formed a fresh portion of cyanide from some of Scheele's acid; this also dissolved in cold nitric acid. It appears, therefore, that the supposed insolubility is at most a matter of proportion, and should not be described as a characteristic of the cyanide.' Mr. Nunneley corroborates this statement. 'I have tried the effect of nitric acid upon cyanide of silver, made with hydrocyanic acid of different strengths, as well as nitrate of silver solutions of different strengths, and I am satisfied that it is, as stated, only a question of proportion. If the precipitate is not very copious, and occasioned by only weak solutions of the acid and salts, then cold nitric acid dissolves it, even if the acid be not of the strongest; but, on the contrary, if the precipitated cyanide be abundant, in large flakes, and made from stronger solutions of the nitrate of silver and hydrocyanic acid, then concentrated boiling nitric acid is required for its solution.' [This statement is correct, and as it affects the value of a very delicate test, on which much reliance is placed, it is not unimportant.]

Mr. Hicks has tested the relative delicacy of the iron and silver tests, and of the odor of the acid. When the tests were added to the solution of the acid the limit in the case of the iron test was 1 minim of prussic acid to ℥iv distilled water: in the case of the silver test, 1 minim to ℥xvj, with which quantity a milkiness was obtained. There was a faint color when the proportion was 1 minim to ℥iv, and a doubtful result with ℥viij and ℥xvj. When watch-glasses moistened with caustic potash and a solution of nitrate of silver respectively, were held over the solution of hydrocyanic acid, sulphate of iron and muriatic acid being subsequently added to the liq. potassæ, the iron test gave 'traces' of its characteristic action with 1 minim in ℥viij, and the silver test a film with as high a degree of dilutation as 1 minim in ℥xxxij. It would, therefore, appear that the modification of the iron and silver tests proposed by Mr. Taylor, possesses, as might be anticipated, greater delicacy than the same tests added to a solution of the acid. Mr. Hicks has also instituted some experiments, with a view of determining the value of this modification of the iron and silver tests as applied immediately, and without previous distillation, to the human body or to the contents of the stomach.

The following is a short summary of Mr. Hicks's experiments:

1. The stomach of a dog, which had been killed by three drachms of prussic acid, after being well washed and allowed to remain sometime in water, was placed in a wide-mouthed bottle, over which a watch-glass, moistened with nitrate of silver, was placed. In ten minutes a white film of the cyanide of silver was deposited on the glass.

2. A cat was killed by 20 minims of Scheele's acid poured into the mouth. Eight hours after death, a small aperture was made in the parietes of the chest, and a watch-glass accurately fitted over it. In half an hour a white margin was formed, and, in an hour, the whole portion wetted with the nitrate of silver was covered with a distinct film of the cyanide, perfectly insoluble in cold nitric acid, but soluble in the acid when boiled. The iron test subsequently applied, gave no indication of the presence of prussic acid. The whole contents of the chest, the stomach, and the lower part of the mouth, were then placed in a bottle, and, on the glasses being applied accurately, the presence of the acid was indicated by both tests. But, on submitting the same to distillation, it could not be detected in the distilled liquor, either by the odor, or by the iron and silver tests.

3. The third experiment consisted in pouring into the stomach of a man, who had recently died, half a pint of porter mixed with half a drachm of Scheele's acid, containing 4 per cent. or 1·12 grains of anhydrous acid. It was laid in a cool place, and examined two days after. The liquid, which had a *beery* smell, but no odor of prussic acid, was put into two bottles, over the mouths of which strips of glass moistened with liq. potassæ and solution of nitrate of silver, were placed. In less than ten minutes, most decided proofs were obtained of the presence of the acid, by the formation of white cyanide of silver in the one and Prussian blue in the other, on the subsequent addition of ferri sulphas and muriatic acid.

*Odor.*—Mr. West, who performed the analysis of the contents of the stomach, and the other fluids taken from the body, in the fatal case recorded by Mr. Nunneley, says, 'I have distilled a portion of the contents of the stomach, at this time, twenty-three days after the poison had been taken, and find the smell, the precipitate with nitrate of silver, and the Prussian blue precipitate. All these are produced, apparently, in the same degree as at first. This case is, therefore, opposed to the common notion of rapid decomposition of the acid.'

Mr. Hicks, in the course of his comments on the first case reported in the Medical Gazette (New Series, No. 14), draws attention to the fact that the odor of the poison was more distinctly perceived on opening the chest than even in the abdomen, and he corroborates this statement by experiments on animals. 'In many instances,' he says, 'where I have destroyed rats, the quantity has not exceeded four minims of Scheele's acid; still, on opening the chest immediately after death, as soon as all the signs of life had disappeared, the odor has been distinctly recognized in that cavity, even when not at all presented in the stomach.'

Experiments on Animals.—The following observations form part of Mr. Nunneley's commentary on the case reported by him in the Provincial Medical and Surgical Journal: 'Out of the great number of dogs, cats, and rabbits, which I have destroyed by the acid, I believe the majority did not shriek.—In some the effect is almost instantaneous,—a few violent struggles, followed by a rest; then a struggle and cry; an involuntary passing of the urine or fæces, and death,—the heart pulsating for sometime after. All other appearances of life have gone, the creature scarcely having moved from the spot. At other times I have seen a dog run about staggering for a minute or more, making attempts to howl, but without succeeding. On the whole, I cannot but think that the instantaneous effects of hydrocyanic acid have been exaggerated, and that the quantity necessary to procure death has been somewhat under-estimated. But different animals will be very differently affected. Thus, birds die almost instaneously. It is sufficient to put the beak of a small bird (a sparrow) into the mouth of a bottle containing only a very small portion of the acid, and the vapor will, in a few seconds, quietly, as though paralysed, without convulsions, destroy it. Mice are very susceptible of its action; rabbits are much more so than either dogs or cats. On the other hand, cold-blooded animals are almost proof against its influence. I have given it to frogs without producing much effect.'

Mr. Hicks states, *inter alia*, that in experiments on dogs and cats he has never known a longer period than forty seconds to elapse before the effects of the poison became visible; and that he has not met with a single instance of death taking place without being preceded by convulsions. He also confirms the statement made by Mr. Blake, that the effects of the acid are not so prompt when it is introduced directly into the stomach, as when its vapor has access to the lungs.

Some useful comments on recent cases of poisoning by prussic acid, on the tests for the poison, and on the treatment, will be found in the Monthly Journal of Medical Science. Much stress is there laid on the value of bleeding from the jugular vein, as a remedial agent, and the practice is enforced by a long quotation from Dr. Lonsdale's treatise.

*Poisoning by Bitter Almonds.*—Dr. Letheby reports the following case, which is given with slight abbreviations. A boy, 13 years of age, was left in charge of his father's shop. He was seen well and cheerful at half past 3 o'clock in the afternoon, but a little before 4, his sister, a child ten years old, found him lying on the floor. She immediately called the father, who found him stretched along the floor upon his back, perfectly motionless and insensible; his face deadly pale; his eyes open and rolling from side to side, and panting for breath. His father raised him into a sitting posture, and then noticed that his limbs, though quite supple, were powerless. In five minutes he was seen by Mr. Henry, a surgeon, who found him still insensible, his countenance placid, but very pale, his eyes half open, fixed, and the pupils dilated, and the lid did not move when the eye was touched. He was breathing slowly, deeply, and at intervals of a minute or so. The pulse at the wrist was gone, though the heart was still beating feebly. Cold water was dashed over the head and face, and an ineffectual attempt was made to administer stimuli by the mouth. About four or five minutes after his arrival the child died, and it is presumed that this must have been about a quarter of an hour from the time of his swallowing the poison. *Post-mortem* examination twenty-four hours after death. Face, placid but pale; eyes, half open; foam at the mouth, having the odor of bitter almonds. The depending parts of the body livid; the lungs congested; the right side of the heart gorged with dark fluid blood; the odor of the poison perceptible in the abdomen, but not elsewhere. The stomach contained twelve ounces and a half of a pulpy, semi-fluid matter, in process of digestion, and having a strong odor of bitter almonds. The internal coat of the stomach, with the exception of some red petechial patches along the greater curvature, was pale. On distilling the contents, with nitrate of silver, one grain of cyanide of silver was obtained, indicating about two-tenths of a grain of pure hydrocyanic acid. On further distillation the volatile oil of the bitter almonds was obtained, free from prussic acid. The points of medical interest in the case were the absence of convulsions; the fact that the boy did not speak after he was seen, that there was no scream, and no evacuation of the stomach, bladder, or rectum. He appeared to die from paralysis of the respiratory muscles, the breathing becoming slower and slower.

Dr. Letheby adds the following summary of the symptoms of poisoning by this agent. 'Those symptoms which appear to be most constant in the case of poisoning by bitter almonds or cherry laurel, are a cadaverous or mortal paleness of the face and body, together with great coolness of the extremities; an almost imperceptible pulse, a slow or panting respiration, and, in the generality of cases, there are no convulsions, but a paralysis, first of the voluntary muscles, and then of the involuntary; after this a profound coma supervenes, and then death. Upon a post-mortem examination, we find nearly the same appearances which hydrocyanic acid produces. The face and highest parts of the body are generally pale, the dependent livid, the countenance is generally placid, the eye half open, glassy, and prominent, the pupil dilated; foam may be adherent to the mouth, and it will smell of the poison; the lungs are greatly congested; the right side of the heart, and the venæ cavæ, gorged with black, uncoagulated blood; the cavities generally have a strong odor of the bitter almond. The stomach may or may not be empty; its contents will always, if recently opened, give out a powerful smell of the oil; the inner coat is generally pale, excepting in one or two places, where there will be a blush of red (I have seen this in three cases of poisoning by oil of bitter almonds), and, altogether, there will be less doubt of the action of the poison than in a case of poisoning by prussic acid, for a greater evidence will be furnished by the odor, which is sure to be present in the stomach.'

*Poisoning by Acetate of Morphia.*—An apothecary's assistant, 24 years of age, swallowed, at noon, fifty-five grains of acetate of morphia, in two ounces of gum water. He then forwarded a letter to his master, informing him of what he had done. The master arrived in half an hour, and gave him, with some difficulty, and without effect, two grains of tartar emetic in an ounce of water, followed by two table-spoonfuls of olive oil. Up to this time the only symptom present was slight giddiness, which soon disappeared, and the lad went for a walk with one of his companions, entered a café, and drank half a bottle of beer. At the end of an hour and a half the lad complained of slight giddiness, a sensation of weight in the limbs, and a slight tendency to sleep. After two hours he was taken to the hospital, put to bed, and made to swallow three grains of tartar emetic, and twenty-four grains of ipecacuanha, in two doses, at an interval of half an hour. Though the operation of the emetics was assisted by tickling the throat, vomiting did not occur, and an œsophagus tube was introduced without effect. At the end of two hours and a half the face, which was previously pale assumed a violet hue; the eyes were injected, and turned upwards and outwards; a viscid perspiration broke out on the skin; the hands and legs grew cold and livid; the lad answered questions only by monosyllables; fell by degrees into a profound sleep; the head fell forward on the chest; the eyelids closed; and the body when left to itself, fell on the side; the legs bent under him;—in a word, he was in the condition of a drunken man. The breathing in the meantime was natural. A pound of blood being taken from the arm, the patient rallied slightly, and complained of not being able to swallow. The pulse, which up to this time was soft and somewhat frequent, became full, hard, and slow, and there was a troublesome itching on the forehead, nose, and lips. This symptom, which began at the end of an hour, was at its height in two hours, and after four hours existed only at long intervals. The treatment now consisted of ammoniacal frictions to the belly and extremities, moxas to the legs, and repeated shaking of the body; but all in vain. The face was distorted; the eyes dull and sunken, and drawn upwards and outwards; and the entire surface of the body was icy cold. At the end of three hours a drachm of tincture of iodine, and the same quantity of hydriodate of potash, in three ounces of water, was given in two doses, at an interval of a quarter of an hour. Immediately after the second dose, the patient rejected what he had taken, but he slept while he was vomiting. Strong coffee was then given every five minutes, which was followed each time by vomiting. Four hours—The coma is still more strongly marked, and the patient can only be kept roused by pinching, tickling and shaking him strongly. He can be made to speak, but will leave a word half uttered. He was again bled to the same extent as before, with the effect of lessening the coma. Four hours and a half—Face violet; the members contracted and stiffened; he is speechless, and cannot be made to open his eyes. Friction of caustic ammonia to the epigastrium, and a blister to each limb. At the end of five hours a third bleeding was performed; strong coffee was still administered, and two blisters were applied to the shoulders. Five hours and a half—From this time the patient began to improve; opened his eyes, though with great difficulty; regained the use of his speech, and endeavored to explain his state to the bystanders. At the end of six hours he recognized the reporter, asked him how he did, inquired whether he did not think him very ill, and expressed surprise that he had survived fifty-five grains of acetate of morphia. At the end of seven hours the symptoms were slowly improving; he could struggle successfully against his tendency to sleep; his ideas were clear; and he could see, as he said, all that was passing, as if through a cloud. At eight o'clock the next morning, sixteen hours after taking the poison, the report was that he had vomited repeatedly during the night, and still continued to do so. On his forehead there was seen a vesicular eruption, which accounted for the itching of the previous day. He still complained of heaviness of the head, and confusion of intellect. On the following day he was nearly well, and left the hospital on the morrow. He still continued to complain of eructations, having the intense bitter taste of the morphia. M. Bonjean regrets that he did not administer a solution of tannin, according to the suggestion of Orfila; and he adduces an experiment to show that the acetate of morphia is completely decomposed by that agent.

*Poisoning by Poppy-heads.*—The Gazette des Hôpitaux (April 15, 1845) quotes from the Echo de la Frontière, published at Valenciennes, the following cases. Three children of one of the faubourgs of Cambrai sucked some unripe poppy-heads, and soon after, on going home, fell asleep. In the middle of the night the mother was roused from sleep by the unusual sound of their respiration, which consisted of a short laborious râle. At the same time, the eyes were found wide open, the countenance pale and haggard, and the body convulsed. A medical man was immediately sent for, who succeeded in saving two of the children. Fruitless efforts were made to introduce an emetic into the stomach of the youngest; deglutition was arrested, the infant foamed at the mouth, and expired four hours after.

*Poisoning by Hemlock* (conium maculatum).—The children of one Duncan Gow, a tailor, of Edinburgh, found what they took for parsley, growing under Sir Walter Scott's monument, and took it home to their father, who, having fasted the whole day, greedily ate the vegetables, with a piece of bread. The quantity taken could not be ascertained, but it probably amounted to several ounces. The history of this case, from the time of the fatal meal, is carefully compiled from the evidence of the several parties who subsequently saw Duncan Gow. The first effect was loss of power in the inferior extremities, unattended by pain. This showed itself soon after the meal. 'His gait, which at that time was faltering, afterwards became vacillating—he staggered as one drunk; at length his limbs refused to support him, and he fell. On being raised, his legs dragged after him; and, lastly, when the arms were lifted, they fell like inert masses, and remained immovable. Perfect paralysis of the inferior extremities was ascertained to exist one hour and a half after the poison was taken, and that of the arms half an hour later.' The complete paralysis of the legs was accompanied by amaurosis; the excito-motory functions were also inferred to be paralysed, from the fact that tickling the armpits failed to produce any movement of the upper extremities. He lost the power of deglutition, and made ineffectual efforts to vomit. 'There were no convulsions, only slight occasional movement of the left leg; and, lastly, both inferior extremities were slowly drawn upwards when placed over the iron of the stretcher. Three hours after taking the poison, the respiratory movements had ceased; the pupils were fixed. At this time the heart's action was very feeble, and ceased altogether ten minutes afterwards. The intelligence remained perfect nearly to the last; two hours after taking the poison he still retained the use of his speech, and a quarter of an hour after that still appeared sensible, though he could not articulate. The post-mortem appearances were, an unusual quantity of fluid blood in the vessels of the scalp, and in the sinuses of the brain; slight serous effusion beneath the arachnoid membrane, and into the ventricles; and numerous bloody points in the substance of the brain. The lungs were 'throughout intensely engorged with dark-red fluid blood. There were a few small clots of blood in the heart. The blood throughout the body, was of a dark color and fluid, even in the heart and large vessels. The stomach contained a pultaceous mass, formed of some raw green vegetable resembling parsley. Its contents weighed eleven ounces, and had an acid and slightly spirituous odor. The mucous coat was much congested, especially at its cardiac extremity. Here there were numerous extravasations of dark red blood, below the epithelium, over a space about the size of the hand.' The intestines and other viscera were healthy, but partially congested. On examining the contents of the stomach more minutely, it was found to consist chiefly of fragments of green leaves and leaf-stalks, and on a specimen being submitted to Dr. Christison, he recognised the *laciniæ* of the *conium maculatum*, or common hemlock. On bruising some of the leaves in a mortar, with a solution of potash, the peculiar mousy odor of conia was at once evident. The conium maculatum was also found growing on the spot from which the leaves had been gathered. Dr. John Hughes Bennet, the reporter of this case, accompanies it by some judicious comments, and shows the striking analogy of the symptoms to those recorded as having followed the drinking of the *Koneion* by Socrates; and justly observes, that that analogy is strongly in favor of the opinion of the identity of the poison swallowed by Socrates and the conium maculatum.

*Poisoning by Arsenic.*—The trial of James M'Kerlie, before the circuit court of Judiciary, at Glasgow, in April, 1845, as reported in the London and Edinburgh Monthly Journal, for June, 1845, comprises some points worthy of notice.

Three persons, a father, his son, and a shopman, partook of the same dish (kail), and some was also given to a dog.—They were all affected more or less severely, but, after frequent vomiting, recovered. The occurrence of vomiting in the dog first excited suspicion as to the cause of the illness being in the food. Dr. Paton, who was first called in, stated that he had tasted the broth, and that it had an acrid, pungent taste, and a powerful effect upon the teeth, from which he concluded that there was arsenic in it. This statement, so much at variance with the experiments of Dr. Christison, deserves to be put on record, though it must be admitted to be quite possible that the acrid, pungent taste might be due to some other substance contained in the broth, and used as seasoning. Arsenic was found in the broth by all the approved tests, and the results communicated in an excellently worded report, drawn up by Drs. Penny and King, of the Andersonian University. James M'Kerlie was proved to have purchased arsenic, under the usual pretence of poisoning rats, and to have been in the kitchen during the cooking of the broth. These, with other suspicious circumstances, induced the jury to find a verdict of guilty, and the prisoner was sentenced to transportation for life.

An interesting and instructive case of triple poisoning by arsenic is recorded by Dr. Bayard. The author's principal object in publishing these cases was to show the care which ought to be taken in medico-legal researches, and the little attention which ought to be paid to mere popular rumors. All the circumstances of the case, and the opinion of the medical men, who saw the deceased during life, were strongly opposed to the idea of poisoning; and it was not till the fifth member of the same family died in the short space of two years, that any suspicions of foul play were expressed. The bodies were then ordered to be examined, when, in three out of five, arsenic was discovered.

*Poisoning by Mercury.* Protochloride of tin as an antidote for corrosive sublimate.—M. Poumet, of Orleans, has examined the value of protochloride of tin as an antidote for corrosive sublimate, by means of a series of well-conducted experiments, and he arrives at the following conclusions:

1. Dogs which are made to take a solution of corrosive sublimate containing $15\frac{1}{2}$ grains, $7\frac{3}{4}$ grains, or $1\frac{1}{2}$ grains of the poison, die, even though allowed to vomit freely.

2. Dogs which are made to take 31 grains of the protochloride of tin in solution recover readily and promptly, even when they are prevented from vomiting.

3. The black precipitate, and the supernatant liquid, the results of the mixture of the salts of tin with a solution of corrosive sublimate, are not poisonous.

4. A solution of the salts of tin, of twice the strength of the solution of corrosive sublimate, when introduced into the stomach, directly after swallowing the poison, neutralizes, instantly and completely, the deleterious property of the salt of mercury, even when the animal is prevented from vomiting.

5. This favorable result takes place, in one-fourth of the cases, when the antidote, instead of being administered immediately, is given a quarter of an hour after taking the poison.

6. The protochloride of tin, then, is an antidote to corrosive sublimate.

7. Would it not be exactly the same with the sulphate and nitrate of mercury? M. Poumet states, that he has reason to answer this question in the affirmative.

In the case of the corrosive poisons, the best antidotes are those which can be most promptly obtained, and as it is much more probable that white of eggs would be at hand in a case of poisoning by corrosive sublimate, than a comparatively rarer agent, the protochloride of tin, it is not likely that this latter substance, though doubtless an antidote to corrosive sublimate, will ever come into use.

*Poisoning by Corrosive Sublimate externally applied.*—John Welch, a druggist, was tried at Worcester, June 18, before Lord Denman, for causing the deaths of two children by a lotion containing corrosive sublimate applied externally for the cure of ringworm. The lotion was applied to the heads of three children. It caused pain, followed by violent inflammation, vesication of the forehead, uneasiness and fever, pain in the head and bowels, and sickness. These symptoms were followed in one of the fatal cases by profuse salivation, ending in sloughing of the gums and tongue. The jury returned a verdict of not guilty, and thus held out an encouragement, scarcely required to unlicensed persons to exercise an art which no previous training has prepared them to understand. No evidence was given as to the death of the other child. The nature of the poison was strongly inferred, but was not proved by analysis.

A case of poisoning by corrosive sublimate, mixed by mistake with sulphate of potass, is related by M. Jean-Baptiste Chevallier. The poisonous mixture was administered by a quack midwife, who was tried before the Tribunal de Premiere Instance de la Seine, for the illegal exercise of pharmacy and the profession of medicine, and involuntary homicide by imprudence. The nature of the mixture was ascertained by chemical analysis.

*Poisoning by Copper.*—The existence of a copper colic in men who work with metal is a subject upon which some doubt has been expressed, its symptoms being attributed to the lead with which it is combined. M. Blondet, in a memoir read before the Academy of Sciences. asserts the reality of the poisonous effects attributed to copper, and describes the colic which it occasions as being subject to remission, characterized by severe pain, increased by pressure, and accompanied by headache, nausea, and diarrhœa, or constipation. The vomited matters are bilious, and the first alvine evacuations are often of a green color. Fever is a somewhat rare accompaniment. In men who are not cleanly, the hair is green; the perspiration of the same color; and the teeth are covered by a grey coating of sulphuret of copper. The prophylactic measures proposed by M. Blondet are the habitual employment of an albuminous drink, and saline purgatives when the bowels are costive. Cleanliness, though not mentioned, is an obvious item of the prophylaxis.

*Poisoning by Zinc.*—M. Blondet, in a memoir presented to the French Academy of Science, directs attention to the symptoms brought on by the fumes arising from zinc used in bronze and brass founding. Heavy pain in the stomach, nausea, loss of appetite, cough, oppression, fixed pain in the head and a sense of tightness across the temples, noise in the ears, which continues during the night, general debility, tetanic stiffness and soreness of the limbs, rigors, trembling of some hours continuance, nightmare, a sensation of swelling, cold sweats preceded by flushes of heat. These symptoms disappear on awaking from sleep, but lassitude and soreness still continue.

*Poisoning by Sulphuric Acid.*—A case of poisoning by sulphuric acid, is reported by MM. Chevallier and Ollivier, which is chiefly interesting from the circumstance of the nature of the poison having been ascertained by an analysis of the clothes, after an examination of some of the vomited matters and of the contents of the stomach had failed—a failure attributed to the free discharge of the poison by vomiting. The post-mortem appearances were very characteristic, and such as to render unnecessary the application of the tests for prussic acid, arsenic, and corrosive sublimate, which the reporters thought it necessary to use.

*Poisoning by Savin.*—An extremely interesting and instructive case of poisoning by this substance, administered to a pregnant female, with fatal effect both to mother and child, occurred in the month of May last, and was the subject of examination in the coroner's court before Mr. Wakley. The poison was identified by the aid of the microscope, the green substance found in the stomach being compared with a portion of powdered savin. Mr. C. Johnson, lecturer on botany at Guy's hospital, stated that on examining a portion of the powder under the microscope, he was enabled to determine it to be the part of a leaf of a plant of the pine genus, by the peculiar arrangement of the glandular apparatus of the leaf, and further, that it was savin by the exact correspondence of the glandular apparatus in question with that of a portion of savin powder examined at the same time. There was the same correspondence between the shape of the extremity of the leaf of the substance found in the stomach and a fragment of savin. The shape of the leaf was widely different from that of the yew, a poisonous plant belonging to the order coniferæ. The contents of the stomach were further identified by their odor. Mr. Alfred Taylor calculated the quantity of savin contained in the stomach at from 25 to 30 grains. The deceased had vomited freely, and brought up a large quantity of green matter which the medical attendant took for bile. The absence of other poisons, whether mineral or vegetable, was ascertained by Mr. Taylor. The jury returned a verdict 'that the deceased, Caroline Hillman, died from the effects of a certain poison called savin, but whether taken for the purpose of destroying life, or procuring abortion, there is not sufficient evidence to show, and that her male infant died a natural death.'

*Poisoning by Sulphate of Quinine.*—M. Desiderio communicated to the February sitting of the Royal Academy of Medicine, the results which he had obtained relatively to the action of this substance, by means of numerous experiments on animals as well as by observations at the bed-side. The effects upon animals are in every respect similar to those observed in the human subject; namely: drowsiness, an indisposition to motion, unsteadiness of gait, dimness of vision, and drooping of the eyelids. An alcoholic solution of morphia produces analogous effects. Hence, when given with sulphate of quinine it increases its action. On the contrary, laurel water counteracts to a certain extent its effects, and may be considered as an antidote. Bleeding is still more efficacious, and digitalis also has appeared to be useful.

*Poisoning by bad Meat acting on vessels of Copper.*—On the 15th April, 1845, the patients, Sisters of Charity, and officials of the Beaujon Hospital, were suddenly, and nearly simultaneously, seized a few hours after dinner with violent colic, frequent and copious purging of bloody mucus, and painful tenesmus. In many instances the symptoms were extremely severe, and they were obviously due to poison. Soups, vegetables, and meat, and the evacuations of the patients, were accordingly submitted to chemical examination, and were found to contain copper. The mere discovery of this poison, however, was not sufficient; for it appeared that many patients who had partaken of the same food escaped, and the meat was forthwith suspected. One of the sisters, the superintendent of the kitchen, recollected that two kinds of meat had been used—a shoulder and a leg of beef—and that those only who had partaken of the latter suffered. Some of the leg still remained; it was cooked, and again eaten; the same symptoms ensued, and the sister nearly lost her life. Nothing of the kind occurred with the shoulder. It was, therefore, evident that the leg was unsound. It appears that on the day of the accident, the contractor had sent to the hospital a quantity of meat, the cellular tissue of which was infected, and evidently diseased; it was accordingly rejected by the superintendent. The contractor immediately killed an ox, which was reported to have just come off a long journey, and consequently in that febrile state which the butchers technically call *heated;* and the portion of it brought to the hospital still reeking, was also rejected. He then appealed to the central administration, which, without previous inquiry, ordered it to be accepted. This was the cause of the symptoms, the presence of copper being thus explained: Meat of this nature generates a quantity of ammonia, which, in spite of the tinning, attacks the copper; hence the formation of ammonuriet of copper, which passes into the broth, and is superadded to the deleterious principle of the meat itself.

*Mode of Operation of Poisons.*—An interesting question connected with the *modus operandi* of poisons has been lately submitted to experiment by M. Andouard, of Beziers, viz: Do poisons, and soluble salts, taken by the mother, reach the body of the fœtus? The following are the conclusions to which we have arrived.

1. Poison and soluble salts reach the fœtus, provided that the death of the mother does not follow instantly on the swallowing of the substance. In this last case, the placenta alone is impregnated with that substance, or, if the fœtus receive it, the quantity is so small as not to be appreciable by analysis.

2. If we have reason to suppose that a pregnant woman has died poisoned, we should not neglect to search for the poison in the placenta, the liquor amnii, and the fœtus.

M. Orfila, in an essay in the Annales d'Hygiéne, devotes himself to the refutation of two errors against which he thinks it necessary to caution those engaged in medico-legal inquiries in cases of poisoning. The first is that of requiring distinct proof that the quantity of poison discovered in the body by analysis is sufficient to destroy life; the second is that we operate more surely if we analyse a small portion of an organ (the liver for instance), than if we employ a larger quantity. It would seem scarcely necessary to occupy upwards of twenty pages in the refutation of two errors so patent as these; but as it has appeared otherwise to this distinguished authority, the subject is here briefly referred to. It must be obvious that if in every case we were to insist on the discovery in the contents of the stomach or the organs of the body of a quantity of poison sufficient to destroy life, many criminals, now justly condemned, would be allowed to escape; and that, on the other hand, if we were to prefer the use of a small portion of the contents of the stomach, or a small fragment of the liver, to a larger quantity of either, we might fail to discover a poison which nevertheless existed in sufficient quantity to be identified by operations on a larger scale. The only case in which operations of a larger scale are to be deprecated is where the reagents we employ are in such quantity as, though apparently pure, to furnish the very poison of which we are in search.

*On Copper and Lead naturally contained in the Human Body.*—M. Devergie, in a paper which forms a rejoinder to a memoir read at the academy, by MM. Danger and Flandin, in which they call in question the assertion of MM. Hervy and Devergie, that the body naturally contains a certain proportion of copper and lead, confirms his original statement by the results of a new series of experiments, performed in conjunction with M. Boutigny. The following are the conclusions at which he arrives:

1. The stomach, the intestines, and all the organs of the economy furnish, on analysis, traces of copper and lead.

2. The proportion in which these metals are found increases with age. It is small in the new-born infant; it is much more considerable in the adult.

3. A chronic malady, during which the food is greatly diminished, appears singularly to lessen the quantity of copper and lead in the organs of the body.

4. This difference confirms the natural supposition that the source of these metals is the meat and vegetables used as food.

5. In all cases the proportion of copper is greater than that of lead.

The theory that the copper and lead naturally contained in the human body is supplied by the food is strongly supported by the fact that copper has been found in wheat, barley, rye, oats, rice, tea, coffee, sugar, sorrel, chicory, chocolate, gelatin, the flesh of the ox, &c ; and that it gives its green color to sorrel, spinach, and gherkins. The use of copper vessels in cookery will also account for the introduction of the metal into the body.

*Action of Charcoal on solutions of Metallic Salts.*—Chevallier has instituted a series of experiments on this subject, to which his attention was directed in consequence of having to examine wine largely impregnated with lead, but which, after being decolorized by animal charcoal, was found to have lost all trace of the metal. The author in the present communication limits himself to the salts of lead, in respect to which he establishes the following facts:—That vegetable charcoal, unwashed animal charcoal, and animal charcoal washed and purified from carbonates and phosphates, not only, as is well known, form with coloring matters, insoluble precipitates, but unite with metallic oxides, so as to separate them from the acids with which they are combined. Charcoal has this effect on aqueous, acetous, alcoholic, and vinous solutions of the salts of lead, though vegetable acts more feebly than animal charcoal. This property which charcoal has of separating the metallic oxides from their solutions M. Chevallier thinks has an important medico-legal application; for in many cases medico-legal authorities prescribe the use of animal charcoal as a decolorizing agent of metallic solutions. M. Chevallier's experiments in 1843, in ignorance of the previous reasarches of Graham.—*Ranking's Half-Yearly Abstract.*

